# Young people’s views about the purpose and composition of research ethics committees: findings from the PEARL qualitative study

**DOI:** 10.1186/s12910-016-0133-1

**Published:** 2016-09-02

**Authors:** Suzanne Audrey, Lindsey Brown, Rona Campbell, Andy Boyd, John Macleod

**Affiliations:** 1 School of Social and Community Medicine, University of Bristol, Canynge Hall, Whatley Road, Bristol, BS8 2PS UK; 2 School of Social and Community Medicine, University of Bristol, Oakfield House, Oakfield Grove, Bristol, BS8 2BN UK

**Keywords:** Research ethics committees, Data linkage, Young people, Qualitative research, ALSPAC

## Abstract

**Background:**

Avon Longitudinal Study of Parents and Children (ALSPAC) is a birth cohort study within which the Project to Enhance ALSPAC through Record Linkage (PEARL) was established to enrich the ALSPAC resource through linkage between ALSPAC participants and routine sources of health and social data. PEARL incorporated qualitative research to seek the views of young people about data linkage, including their opinions about appropriate safeguards and research governance. In this paper we focus on views expressed about the purpose and composition of research ethics committees.

**Methods:**

Digitally recorded interviews were conducted with 48 participants aged 17–19 years. Participants were asked about whether medical research should be monitored and controlled, their knowledge of research ethics committees, who should sit on these committees and what their role should be. Interview recordings were fully transcribed and anonymised. Thematic analysis was undertaken, assisted by the Framework approach to data management.

**Results:**

The majority of interviewees had little or no specific knowledge of ethics committees. Once given basic information about research ethics committees, only three respondents suggested there was no need for such bodies to scrutinise research. The key tasks of ethics committees were identified as monitoring the research process and protecting research participants. The difficulty of balancing the potential to inhibit research against the need to protect research participants was acknowledged. The importance of relevant research and professional expertise was identified but it was also considered important to represent wider public opinion, and to counter the bias potentially associated with self-selection possibly through a selection process similar to ‘jury duty’.

**Conclusions:**

There is a need for more education and public awareness about the role and composition of research ethics committees. Despite an initial lack of knowledge, interviewees were able to contribute their ideas and balance the rights of individuals with the wider benefits from research. The suggestion that public opinion should be represented through random selection similar to jury duty may be worth pursuing in the light of the need to ensure diversity of opinion and establish trust amongst the general public about the use of ‘big data’ for the wider public good.

## Background

### Purpose and composition of research ethics committees

Medical research, in particular research that involves the secondary use of medical records, is regulated by a range of independent oversight bodies whose role is to ensure that only research that is both ethical and compliant with all relevant legislation and official guidance is permitted. These include: research ethics committees (RECs) with a general role of scrutiny towards research proposals in relation to recognised ethical standards which include respecting the dignity, rights, safety and well-being of the people who take part; bodies with a gatekeeping role in relation to requests to access specific research data resources [1]; bodies with a remit to ensure compliance with the legislative framework around secondary use of data from medical records in research [2], and; study level ‘patient and public involvement’ committees, which include those providing advice on research ethics [3], management and data usage [4].

International and national guidance has been provided about the purpose and composition of such bodies [5, 6]. The main responsibility is to protect research participants, but potential risks and benefits for the wider community are also taken into account. Committees have the authority to approve, reject or stop studies or require modifications to research protocols. Specific tasks include: identifying and weighing up the risks and potential benefits of research; evaluating the process and materials to be used for seeking participants’ informed consent; assessing recruitment procedures and any incentives or coercive behaviour towards participants; considering measures for patient or participant involvement in the research design, and; evaluating risks to participants’ confidentiality. In some circumstances committees make judgements around competing priorities, particularly in circumstance where seeking individual consent is either impractical or likely to introduce bias. These priorities may include balancing the imperative to minimise risk of inadvertent disclosure of identifiable sensitive information against the priority to use data effectively and efficiently to increase the health of the public.

Established guidance states that membership of oversight committees should include sufficient expertise and competence to undertake the committee’s primary purpose [5, 6]. For example, in the case of RECs, expertise and experience is required to enable the rationale, aims, objectives and design of research proposals to be reconciled with the dignity, rights, safety and well-being of potential research participants. Individuals with relevant medical or scientific expertise are required to assess the procedures to be employed, potential consequences for participants, and the scientific validity of the study design. But some risks and benefits, for example relating to cultural or legal issues, may be more easily identified and assessed by members with other qualifications or professional expertise. As well as containing ‘expert’ members, RECs are generally expected to include ‘lay’ members who reflect public opinion and are able to assess the suitability of the information that will be given to prospective participants as part of the informed-consent process. Social diversity (including factors such as age, gender, ethnicity, socio-economic status and disability) should ideally be reflected in the committee so that decisions are not dominated by a single perspective or vested interests. In practice, however, this diversity of membership and perspectives may be difficult to achieve. It has been argued, for example, that a preponderance of institutional or scientific members may result in an atmosphere that feels intimidating for community representatives and other minority members [7].

### Data linkage

An important ethical issue for the research community, and wider society, is the secondary use of data that is routinely collected for administration purposes. Although not acquired for the purpose of conducting health research, these data can be valuable for epidemiological research for several reasons. These data may be easier to access and more accurate than, for example, information collected in self-report questionnaires [8]. Data linkage can be a cost efficient way to follow up participants, and can contribute information that would otherwise be missing. It can also inform statistical methods for dealing with missing data in study samples [9]. Information from different sources, about the same people, can help to establish connections between health, behaviour, the environment and other risk factors [10–13]. The benefits gained from data linkage tend to increase with the quality and quantity of data available.

The potential to derive benefits from data linkage needs to be balanced against the risks to participants including, for example, the threat to privacy. Although records are usually identifiable at the point of linkage, many of the concerns about privacy can be addressed if anonymized linkage technologies are used [14–16] or if suitable governance models can be established using frameworks such as ‘Data Safe Havens’ which provide protections equating to effectively anonymised data where configured appropriately [17, 18]. However, Laurie et al. suggest that protection of privacy is never absolute, and this should be acknowledged [19]. They argue that responsible data linkage requires a proportionate and adaptable approach, with public and stakeholder engagement, and mutual agreement about the tenets of good practice. Such an approach is likely to be facilitated by ethical review that is proportionate to the scale and complexity of the research proposed, and includes public and professional opinion in reaching decisions.

### ALSPAC and PEARL

Avon Longitudinal Study of Parents and Children (ALSPAC) is the largest birth cohort with detailed biological and behavioural data from before birth to early adulthood in the world (www.alspac.bris.ac.uk). ALSPAC recruited women resident in and around the city of Bristol, UK with expected dates of delivery between 1st April 1991 and 31st December 1992. The total sample size included 15,247 pregnancies of which 14,701 babies were alive at 1 year of age [20]. Participants have been regularly assessed since then, mainly through questionnaires and clinics and analysis of biological and genetic samples. The study website describes the resource in more depth and contains details of all the data that is available through a fully searchable data dictionary (http://www.bris.ac.uk/alspac/researchers/data-access/data-dictionary/).

Within the main ALSPAC study, the Project to Enhance ALSPAC through Record Linkage (PEARL) seeks to gain consent for linking ALSPAC participants to routinely-collected social and health data, and establish appropriate methods for this data linkage [21]. PEARL was established to enhance the ALSPAC database, improve strategies to reduce bias resulting from missing data, and enable prospective follow-up that minimises the burden to participants and is cost-efficient. PEARL incorporated qualitative research to seek the views of young people about data linkage, including their opinions about appropriate safeguards and research governance. In this paper we focus on the qualitative data relating to the purpose and composition of research ethics committees in monitoring medical research, particularly where this involves data linkage.

## Methods

### Recruitment

The recruitment strategy for the qualitative study aimed to include young people with differing backgrounds and levels of participation in the ALSPAC study. An initial random sample was supplemented by a purposive sample based on random selection within specific sub-cohorts: i) low socio-economic status i.e. living in areas within the two most deprived quintiles as defined by the Index of Multiple Deprivation, ii) history of low participation in ALSPAC, and iii) ‘care cases’ highlighted by a study administration flag to indicate sensitivity in relation to health or family circumstances). One of the authors (AB), who has a trusted role within ALSPAC, undertook the purposive sampling using linked education records and information derived from the ALSPAC administrative database, and operating within the Data Safe Haven. These participants were blinded to the purposive sampling.

Potential participants were sent an information leaflet and a letter of invitation, including a reply slip and pre-paid envelope to return if they were willing to be interviewed. One reminder was sent. Those who expressed an interest were contacted by the researcher (LB) who telephoned to arrange a face-to-face interview. Before the interview, written consent was obtained. The ALSPAC Ethics and Law Committee (Reference number E200905) gave ethical approval for the study.

### Interviews

Participants chose whether to be interviewed at home, in a public place such as a café, or in a private room at the University of Bristol. One researcher (LB) conducted all the interviews, which were digitally recorded. The interview topic guide was shaped by relevant literature, expertise within the research team, and issues that arose as interviewing progressed. Explanations were provided at key points during the interview, including sketching and discussing diagrams, to aid understanding of key issues relating to different types of consent and record linkage.

On the topic of research governance the topic guide included the following questions: Do you think that medical research is monitored and controlled; Who do you think does it; Is it important that research is controlled; Who do you think should do the monitoring; What kinds of safeguards do you think are in place to make sure the researchers are properly qualified and that the research they are doing is good; Have you heard of ethics committees; What do you think they might do; When considering this type of research (on medical records, data linkage) what kind of things do you think they should think about; Who do you think should sit on these committees?

Prompts and explanations were provided, depending on the needs of the individual interviewees. Where participants had not heard of research ethics committees, a brief description was provided of the oversight arrangements related to medical research in general and research involving the secondary use of administrative data in particular.

### Analysis

Interview recordings were fully transcribed and anonymised by removing any information that might identify participants. Framework methodology was used to aid thematic analysis of the large qualitative dataset [22, 23]. One researcher (SA) created a primary chart with a row for each participant and columns containing original text about monitoring research and research ethics. This chart was streamlined as analysis progressed. Key terms and phrases were coded and retained, while repetition and extraneous text were removed. For this paper, the process of summarising and coding the data focussed on three main issues: participants’ knowledge about whether and how medical research is monitored; understanding of the role of ethics committees, and; views about the composition of ethics committees. Separate charts were compiled for each of these three broad areas, and similarities and differences in the views of participants were explored. An illustrative section of a chart is given in Table 1. A second qualitative researcher (RC) examined the charts to check interpretations which were discussed and agreed by all authors.

**Table 1:** Section of chart relating to the role of ethics committees

ID	Text	Code
1	I think it’s very important that they [researchers] have like a standards and stuff and I mean their morals I guess, if they’re, they’re important but as long as they’ve got that, a standard, and there’s somebody checking that they keep to that standard, like um a sort of separate body, you know, if that makes sense? A sort of um governing body, that’s it. That makes sure that they keep to those standards and that sort of, you know, ethics code, I guess. … Um make sure things are being used in the right way, make sure things you know are ethically used … how important is the research … [if] other people’s information needs to be used, whether it does need to be used, whether there’s another way of doing it … whether somebody could be perhaps at risk or something if they do use the information and stuff like that.	Standards Morals Separate body Right way Importance Purpose Risk
2	Making sure obviously that you’ve got a fair sample, of a fair, um that every group, every, all types of people are represented within the study	Fair sample Representative
3	Um it’s probably a good thing, check everyone’s doing everything right … maybe what kind of records they’re looking at. Um obviously they’re going to be quite personal things if it’s stuff like medicals stuff, um but I don’t know. Maybe checking that people have given their consent and that all, that the organis- the research organisation has tried to get that consent, that kind of thing.	Right way Type of records Personal Consent

Rows and columns continued to include all participants (*n* = 48)

## Results

### Participants

For the main study, 55 young people were interviewed but in seven interviews there was no discussion of ethics committees: where time was short, or a young person was struggling to understand concepts, priority was given to explaining and discussing data linkage and consent procedures. This paper focusses on the responses of 48 young people of whom 75 % had participated in the ALSPAC study from birth, 92 % were White British and 58 % were female. Just over half (54 %) of the interviewees lived in more disadvantaged areas by IMD score, and 10 % were disabled or had a long-term illness (Table 2).

**Table 2:** Characteristics of participants (*n* = 48)

	Number
Age	
17 years	11
18 years	29
19 years	8
Gender	
Female	28
Male	20
Ethnicity	
White British	44
Mixed	2
White other	1
Declined to say	1
Employment	
Student	35
In work	6
Apprenticeship	1
Unemployed	6
Participation in ALSPAC	
Regularly since childhood	36
Infrequent since childhood	5
No previous involvement	7
IMD score	
(most deprived) 1	10
2	16
3	6
4	6
(least deprived) 5	10
Disability/long term illness	5

### Knowledge of ethics committees

The majority of interviewees had little or no specific knowledge of ethics committees. Nevertheless, there appeared to be an implicit assumption that there was a system for checking the activities of researchers:

Well I’ve always assumed there was a system … Because um after 10, 20 years (*laughs*) you almost expect there to be some kind of system in, in place. (Interview 17)

One young person, who was not aware of ethics committees, felt information should be available for those who were interested.

Maybe people should be made aware. I mean, I don’t think it’s necessary to throw loads of information about the whole runnings of everything at people because one they are not going to read it and it might just sort of annoy them, like ‘Look how much I’ve got to read or be told’. And, you know, they might not really, maybe not even understand or really care either way. But yes, the option should be there for people to find out about how it’s all done. (Interview 11)

Of the 11 young people who said that they had heard of ethics committees, six were unclear where they had gained the information and five indicated they had learned about ethics committees at school. For example:

We did it in VIP … Values in Practice … it’s just some rubbish thing they make you do at school (*laughs*). It’s really rubbish … it’s like a combination of geography and, English, RE and some other stuff thrown in and it’s just, it talks about things, you know, society and stuff and about, you know, what’s right, what’s wrong um about ethics and stuff and that’s where I heard about it. (Interview 1)

Through biology … Not tons, just yeah, just that they exist and they’re supposed to be there for our good. (Interview 37)

### The role of ethics committees

Following a brief summary by the interviewer of the purpose of ethics committees, the young people were asked to consider the value of this role. Only three respondents suggested there was no need for ethics committees to scrutinise research. One respondent (Interview 30) was unusual in consistently indicating that he had no concerns about anyone accessing any of his personal data without his consent. Other reasons given for not consulting ethics committees were the difficulty of monitoring what researchers were doing in practice, and the potential to restrict the research process.

Well you don’t necessarily have to do it [*consult an ethics committee*] cos they don’t know that you’re necessarily actually doing what you say you’re doing anyway … I mean, I’m sure you are but I’m just saying like you could be doing something completely different that they don’t know about. (Interview 28)

They [*researchers*] should just do what they say. It shouldn’t need to be checked up on because they’re doing research that’s going to help … I think that’s [*ethics application*] just too much hassle to the point where you might just not bother doing the research. (Interview 29)

The difficulty of balancing the potential to inhibit research against the need to protect research participants was specifically acknowledged:

It [*ethics application*], well it’s beneficial. It’s just if it will, is it too strict? Will it actually stop progress being made too much? (Interview 32)

It [*ethics application*] might put some researchers off trying to do studies but … the usual ethical guidelines [*should be followed*] if it’s going to, yeah, like if it [*the research*] could have any really bad consequences afterwards like socially or politically. (Interview 42)

It was suggested that ethics committees could offer guidance to researchers. This might be telling researchers “whether it’s OK” (Interview 31) or “you’re doing it wrong” (Interview 9) to avoid potential scandals:

You’ve got to be kind of over-protective with stuff like this because some awful case will happen and everyone will kick up a stink. You only need one awful case to get into the press. (Interview 45)

The majority of views expressed about the purpose of ethics committees related to two priorities: monitoring the research process and protecting research participants.

#### Monitoring researchers

It was suggested that researchers need an external body to ensure they adhere to the required standards for ethical research:

As long as they’ve got that, a standard, and there’s somebody checking that they keep to that standard, like um a sort of separate body, you know, if that makes sense? A sort of governing body, that’s it, that makes sure that they keep to those standards … make sure things are being used in the right way … how important is the research … whether there’s another way of doing it … whether somebody could be perhaps at risk or something if they do use the information and stuff like that. (Interview 1)

In particular, researchers should not feel they can do as they please:

Otherwise you would just like do anything … you can get a bit selfish, not on purpose but like if you really want to do something. You wouldn’t, kind of like, also consider other options. (Interview 4)

Examining the purpose of the research was considered important:

Just to make sure you’re not like just doing it to be nosey or whatever, just to see that you’re actually doing it for like a, like a proper reason. (Interview 25)

I’d say there would probably have to be an overruling body … Then I mean if it was something that they don’t agree with, then they might say “No” straightaway. But if it’s something like a cure for cancer, and you’re getting an answer, then they might say “Well you can keep going with this but, once you get to a dead end, then you stop”. And maybe they, they’d have to have, sort of first, legally they would be the person who would be able to stop it if it needed to be stopped. (Interview 38)

This monitoring process was also linked to the efficient use of resources “to like make sure that they do it properly and don’t waste the money and waste people’s time” (Interview 10).

I’d want to know that there’s, there’s been something that mediates it all … we’re not exactly the poorest country in the world but I’d say that, well I wouldn’t want to waste money on stuff that’s not necessary. (Interview 16)

They can decide if it’s worth doing or not … like how advantageous it would be to the general public and like how much it would cost and all that. (Interview 43)

#### Protecting research participants

A primary role for ethics committees was acknowledged to be the protection of research participants:

Got to make sure that the participants aren’t going to be harmed in the study… make sure the information’s going to be confidential, make sure that the results aren’t going to have negative effect on society, um got to ensure that the participants have already had, given like informed consent, or general consent at least … they’re some of the ones that I’ve learnt about and they are the ones that I would think are more important ones. (Interview 20)

Obviously everybody has a different level of things that they’re comfortable with … you can’t go in guns blazing, saying things that are gonna upset the person you’re trying to get information from. (Interview 37)

Different types of research were thought to require different levels of protection for research participants, and there were concerns about acquiring data without the knowledge or consent of the participant:

I think in this [*interview*] situation I think it [*ethical approval*] is a bit over the top like. Sort of like we’re not giving you blood or anything (*laughs*) like you’re just asking me questions … but I think for other things like medical records and stuff I think it will definitely be more important … Like if there’s something wrong with you, like other people shouldn’t really be able to know that as easily … in that situation like it would be someone going to get information and I’m not there. Whereas here I’m choosing what I can, what to say to you, but I can decide what to say. (Interview 15)

An important aspect of the role of the ethics committee was to have the “best interest of other people at heart … then they’d make the decisions that’s like, like for everybody” (Interview 50). Consideration of the role inevitably entailed discussion of who sits on ethics committees and contributes to such decisions.

### Composition of ethics committee

#### Expertise

Relevant research expertise was considered important including “people that have done research before, so like they know what they’re doing and what they need to do” (Interview 6) and “someone who knew about the area of research to work out whether it was feasible or not” (Interview 32).

But other types of expertise were also considered important:

I think probably like judges to be honest actually. Cos obviously they make quite good judgements (*laughs*). Not all the time but sometimes they do, um perhaps doctors and stuff because you know I guess they know stuff that was important about research and stuff that’s important. Maybe, you know, a police person should be on there because again with like you know safety-ness and stuff, they’d know about that … and maybe like accountants and stuff cos you know they seem to be everywhere so they’re quite important. (Interview 1)

Other experts included: “doctors, psychologists” (interview 14); “a government official maybe, just for like ‘Is it right with what the government wants to be doing right now?’ sort of thing” (Interview 38); “a legal representative” (Interview 15); “human rights person” (Interview 44), and; “philosophers” (Interview 11). This mixture of expertise was described by one participant as “a group of geeks basically (*laughs*)” (Interview 26).

#### Balance and representation

Although expertise was considered important, it was also felt that wider representation was needed to minimise the influence of vested interests.

I’m thinking there’d be possibly some representatives from just any job, any sort of people maybe, just to say how they’d feel if they were being asked those questions because a scientist will have a vested interest in whether or not some research will go through won’t they? So you’d need um, all sorts of people really. Lots of people who know what this is about, you know, scientists, researchers themselves, ethics people but also some people who wouldn’t really have a particular interest either way. (Interview 11)

It was considered important “to get like a fair representation of everyone’s opinions on research” (Interview 42). This could include “poor people and rich people and middle class people” (Interview 25) and “say you have got one … far right conservative, you should even it out with a liberal mind on the other side so that it is not just too one sided” (Interview 5).

I guess you’d want someone from a different like from an ethnic background maybe, maybe someone from a wealthy background. You don’t want all the same type of people otherwise it’s just not going to be able to work is it, that’s not really what a committee’s about … you don’t want people under the same impression, do you? You want people with different views I guess. (Interview 18)

Not necessarily just race and gender and age and things, you probably want people from different job backgrounds as well. Like people, you wouldn’t want just people who sat in an office all day, you certainly want people like shop keepers and um people from like who have more hands on jobs as well because, particularly don’t want just educated people I think cos then, because that, because I think the opinions of some is, is just the opinion of a small group of people not the opinion of everybody. (Interview 24)

This wider representation of views might be “in some proportion to the sort of amounts of that type of person in the UK” (Interview 33), and would aim to ensure that “the collective thought that they’ve all come up with is a fair one” (Interview 45).

#### Public representation

To support this balance of opinion, and especially to represent the general public, it was suggested that participation in ethics committees should be a “bit like jury duty in a way” (Interview 12), “to have a variety of opinions … some people such as jury duty … a lot of different people” (Interview 9).

It should be like a jury … it’s like a group of people and like you get, you get told from a random selection of people. Not like a set group of people each time. And then it'd be like, they decide … it’s really hard to choose who should sit on it cos different people have different opinions, don’t they, about what’s ethical and what’s not ethical and what’s right and what’s not right and what should be done. So it needs to be like how we choose a jury like, random, like different groups of people rather one set of type of person. (Interview 19)

The contribution of these members was not expertise but to represent public opinion:

Because otherwise the researchers are a bit like a, a clique again. What, what their opinion is might not be the opinion of everybody. So you need to ask the opinion of people who aren’t researchers like Joe Public, which is why, whether or not they feel comfortable about what things they’re researching. (Interview 24)

Maybe just one normal person. Like, yeah like one um person who would say like “Oh I don’t think people would be willing to take part in that.” (Interview 4)

These members might include “a representative of one of the people, like one of the people that would be studied” (Interview 15), which was thought important because “if something is gonna affect me, I’m gonna wanna have a say in it” (Interview 37). Alternatively, it might be “a few kind of like average people” (Interview 43).

It was felt that, for these members, specific expertise was not needed “so long as they all know what’s ethical and what’s not that’s fine” (Interview 21). One respondent suggested “give them a test and see if they’re of like sound of mind and stuff and then … they’d all have different bits of knowledge they could bring to the like overall opinion” (Interview 43).

“They probably should know, and probably know better than a lot of the others, because they’ll be the kind of people that, whose information would be used probably. So then, if they agree that it’s all right then probably, I don’t know, the majority of people would feel that their stuff’s OK.” (Interview 47)

## Discussion

### Balancing interests

The PEARL study was established specifically in relation to data linkage to enhance ALSPAC. However, the issues raised are pertinent to the increasing demand within medical research for secondary use of data which may entail linking data from different sources into larger datasets or using data for purposes not known by the original study participants [24]. Similar issues also arise in relation to genetics and genomics research where there have been calls for consensus on the appropriate use of archived data and consent procedures for future unspecified research [25]. More broadly, some of these issues – particularly representation in committee membership– tie in with Patient and Public Involvement initiatives such as INVOLVE [26].

It is argued that research participants require adequate protection while the cost, time and administrative burden for researchers should be minimised to enable wider society to reap the benefits of opportunities to exploit existing data using new innovations in informatics and particularly ‘data linkage’ infrastructure and methodologies. This may be a difficult balance to achieve and there are concerns that if regulations are too stringent they will hinder, or make impossible, research that contributes to improving health outcomes and health systems performance [27]. The Department of Health in the United Kingdom (UK) has suggested that ‘the duty to share information can be as important as the duty to protect patient confidentiality’ (p.21) [28]. The importance of these issues was acknowledged by young people in the PEARL qualitative study.

### The role and composition of ethics committees

Although three-quarters of the interviewees had been involved in the ALSPAC study from birth, the majority indicated they had little or no knowledge of ethics committees. This would suggest that far more needs to be done to educate and inform young people, and the wider general public, about the purpose of ethic committees. However, once given an outline of the role of ethics committees, the young people contributed a range of interesting and relevant ideas about their purpose and composition.

Casteleyna et al. [24] suggest ethics committees could play a key role in assessing competing needs and building trust in science amongst (potential) study participants and the wider community. They argue the perceived tension between privacy and data sharing within health research should not be overemphasized, as studies have highlighted public willingness to participate in research with a view to advancing scientific knowledge in the interests of all. This is supported by previous PEARL research which showed participants felt positively about health related research, believing in the importance of participating in studies which were conducted for the benefit of the wider public, although this did not entitle researchers to do whatever they liked with participants’ data [29].

van Veen [30] suggests the ethical rationale of research involving data sharing can be based on the principles of citizenship and solidarity. He argues a good research governance framework should not establish rules but principles which provide flexibility, and a fair balance between the interests of all stakeholders. Such research governance, he argues, should be developed by researchers together with patients who, as 'biosocial citizens', are the natural allies of researchers against the 'paternalistic attitudes' of some ethicists and regulators.

Burton et al. [17] argue data usage, whilst being consistent with formal ethical and legal requirements, must also be responsive to emerging issues raised by new research and ethico-legal developments. This responsiveness depends upon inclusive structures, representing all stakeholders including patients or research participants, that are able to deal with issues in a timely way whilst ensuring the public and research participants have sufficient trust and confidence to provide data upon which research and healthcare development depend.

A useful distinction can be made between lay and community representation on ethics committees [31]. Lay representation refers to members with no scientific or medical background but who usually have professional expertise that they contribute to the deliberations of the committee, for example lawyers, economists or theologians. They may be quite different from the patients or participants being researched, although lay and community representation are often conflated. Interestingly, the young people in the PEARL study did appear to differentiate between health researchers, other experts, and “ordinary” or “normal” people and felt there was a role for each in the deliberations of ethics committees. This has been implemented within ALSPAC, where membership criteria of the ALSPAC Ethics & Law Committee [32] distinguishes between clinicians, professionals, lay members and study participants (who should comprise half the membership of the committee). Both ALEC, and more broadly ALSPAC, are advised by the ALSPAC Original Cohort Advisory Panel; a committee of study participants who advise on research management issues. However, in cases involving the use of health service data, or the collection of data via direct assessment, the ultimate decision making lies with NHS committees, and are therefore beyond the influence of study patients/participants.

Dyer [33] argues, while the public once trusted experts simply because of their expertise, the relationship in late modernity is more complex and contested. He favours greater public involvement in ethics committees but suggests, if there is a lack clarity about what they are being asked to contribute and how, there is a risk of wasting peoples’ time and endangering trust between experts and non-experts. Given their potential role in promoting greater openness and trust, he speculates that members of the public should be selected as they are for jury service where their role would be to assess the elements of research concerned with social values [33]. Several young people in the PEARL study also suggested that public participation in ethics committees might be similar to jury duty. In England and Wales, the public retain high levels of trust in the jury system [34] and this may be an idea worth pursuing in the light of the need to establish trust amongst the general public in the use of ‘big data’ for the wider public good.

### Strengths and limitations

The recruitment strategy resulted in a large sample for a qualitative study of this kind, and this enhanced diversity. Quotations included in this manuscript were chosen because they illustrate particularly well the specific points made. The views expressed are from a range of participants: of the 48 young people who discussed ethics committees, 29 are directly quoted in this manuscript, either in longer quotations or phrases/words. Of these 29 young people: five had a disability or long-term illness; seven were unemployed; five had never participated in ALSPAC activities despite being eligible, and four had only partially participated; nine were in the lowest socio-economic group and five were in the highest. This suggests that the young people from the purposively chosen sub-groups did not have difficulty engaging with the topic and expressing their opinions.

Theoretical saturation was achieved, where additional sampling did not lead to new information relating to the research questions. Some responses show a level of understanding and maturity that might not have been expected from young people who, by their own admission, had previously not given much consideration to the topic. The majority of participants had been engaged with ALSPAC since birth and may have different perspectives about research from young people who are not part of a research cohort. Nevertheless, they expressed a range of views and raised interesting issues. We should not, therefore, assume that young people are unable to understand these topics; the interviews illustrate that young people can make a valuable contribution to discussions about research ethics and the deliberations of ethics committees.

## Conclusion

This study suggests that there may be a need for more education and public awareness about the role and composition of research ethics committees. Nevertheless, despite expressing an initial lack of knowledge about ethics committees, the respondents demonstrated an apparently sophisticated understanding of the relevant issues and were able to contribute their ideas and to balance the rights of individuals with the wider benefits from research. In terms of the composition of ethics committees, they expressed the need for expertise but also emphasised the importance of considering wider views from members of the general public. Respondents also appeared to recognise the potential for bias if committee members were predominantly self-selected. Some respondents advocated recruitment onto ethics committees through a system similar to jury duty, to ensure diversity of opinion and enhance trust amongst the wider public.
